# KS-NailMel-1: a novel cell line of nail apparatus melanoma

**DOI:** 10.1007/s13577-025-01242-7

**Published:** 2025-05-28

**Authors:** Takamichi Ito, Yuka Tanaka, Keiko Tanegashima, Kiichiro Nishio, Hiroki Hashimoto, Toshio Ichiki, Fumitaka Ohno, Yumiko Kaku-Ito, Takeshi Nakahara

**Affiliations:** Department of Dermatology, Graduate School of Medical Sciences, Kyushu University, 3-1-1 Maidashi, Higashi-ku, Fukuoka, 812-8582 Japan

**Keywords:** Nail apparatus melanoma, Acral melanoma, Cancer cell line, Cancer model, Chemosensitivity

## Abstract

Nail apparatus melanoma (NAM) is a specific type of cutaneous melanoma that develops in the nail apparatus of the hands and feet. The prognosis for metastatic NAM is poor due to a lack of fully effective systemic therapies. However, the difficulty in obtaining a NAM model has hindered basic research aimed at discovering effective treatment strategies. In this study, we established a NAM cell line, named KS-NailMel-1, from a primary tumor located on the nail apparatus of the left ring finger of a 68-year-old Japanese female. The cells were successfully maintained for over 9 months, exhibiting a doubling time of 38.6 ± 1.94 h. KS-NailMel-1 displayed consistent growth, spheroid formation, and invasiveness, and was confirmed to be identical to the original tumor through short tandem repeat analyses, whole-exome sequencing, and immunohistochemistry. Western blotting of the cells demonstrated the protein expression of NECTIN4, which has recently attracted attention as a potential therapeutic target for melanoma. The KS-NailMel-1 cell line represents a valuable resource for basic and preclinical research on NAM, deepening our understanding of the tumor characteristics and facilitating the development of treatment strategies for this rare form of cancer.

**Supplementary Information:**

The online version contains supplementary material available at 10.1007/s13577-025-01242-7.

## Introduction

Melanoma is a lethal type of malignant tumor that primarily affects the skin, and its incidence is rising worldwide [1, 2]. Nail apparatus melanoma (NAM), which constitutes 0.7–3.5% of all cutaneous melanomas, is a distinct form of melanoma that occurs in the nail apparatus and is part of the acral melanoma subgroup [3–9]. While acral melanoma, including NAM, has similar incidence rates across different ethnic groups, it represents a larger proportion of melanoma cases in individuals with darker skin, as non-acral melanomas are less common among people of color [10, 11]. Specifically, non-acral melanomas account for approximately 0.18–2.8% of cases in Europeans, 10–23% in Asians, and 25% in African Americans [12]. NAM is primarily treated by general protocols for cutaneous melanoma, but there is limited evidence supporting this. Notably, acral melanoma has a different genetic profile from non-acral melanoma, and recent data suggest that NAM may also possess a distinct genetic background [13–17].

Complete removal of the tumor in its early stages is curative; however, invasive NAM increases the risk of lymph node involvement or distant metastasis [18–20]. Therefore, early detection and therapeutic intervention is crucial [21–23] and systemic therapies are necessary for unresectable or metastasized NAM. Although immune checkpoint inhibitors (ICIs) and BRAF/MEK inhibitors have transformed melanoma management and significantly improved patient survival, treating unresectable NAM remains challenging due to the low frequency of *BRAF* mutations and resistance to immunotherapy [19, 24–34]. *BRAF* mutations are found in only about 10% of NAM, and anti-PD-1 monotherapy has shown effectiveness in only a small subset (8.6%) of patients with metastatic NAM [13–15, 17, 21, 25, 35–38]. There is thus an urgent need to develop novel therapeutic strategies for unresectable NAM.

Cancer cell lines are vital for conducting basic and preclinical research, enabling functional analyses [39, 40]. However, cell lines of NAM that can be cultured and maintained for long periods in conventional in vitro systems have rarely been established [41]. In this study, we established a NAM cell line named KS-NailMel-1 from a patient’s primary NAM lesion. The cells were positive for melanoma markers and showed chemosensitivity to anticancer drugs. We also investigated the expression of genes related to apoptosis/survival, proliferation, migration, and invasion. Additionally, we explored the protein expression of HER3 and NECTIN4, as they are potential therapeutic targets for melanoma and various other skin cancers [42–49]. This cell line represents a promising resource for basic and preclinical research on NAM.

## Materials and methods

### Ethical approval

This study was conducted in accordance with the principles of the Declaration of Helsinki. The Ethics Committee of Kyushu University Hospital in Fukuoka, Japan, approved the experiments (approval number 21050–00, approved on November 10 th, 2021). Written informed consent was obtained from the patient prior to her inclusion in the study.

### Immunohistochemistry (IHC)

Immunohistochemical analysis of the patient’s NAM lesion was performed using formalin-fixed paraffin-embedded tissue archived at Kyushu University Hospital. The tissue was sliced into 4-μm-thickness and stained as described before [50, 51]. The antibodies used are summarized in Supplementary Table S1. The chromogens used were 3,3′-diaminobenzidine tetrahydrochloride (725,191; Nichirei Biosciences, Tokyo, Japan) and FastRed II (415,261; Nichirei Biosciences). Stained samples were observed under a Nikon ECLIPSE 80i microscope (Nikon Co., Tokyo, Japan).

### Establishment of a novel cell line from the patient’s NAM lesion

Surgically resected NAM lesion of a 68-year-old Japanese female was used to establish a cell line. The patient first noticed a black subungual patch on her left ring finger, but did not seek treatment at that time. Ten years later, the patient came to us because the size of the patch increased. We performed amputation surgery on the affected finger. A part of the obtained primary lesion was minced and digested in DMEM (D6429; Sigma-Aldrich Co., St. Louis, MO) containing 10% fetal bovine serum (FBS, 175,012; Nichirei Biosciences) and 1 mg/mL collagenase type I (35–17604; Fujifilm Wako Pure Chemicals, Osaka, Japan) at 37 °C for 60 min. The cells were suspended in Endothelial Cell Basal Medium 2 (C-22111; Takara Bio Inc., Kusatsu, Japan) and seeded into collagen type I (Cellmatrix Type I-P; Nitta Gelatin, Osaka, Japan)-coated dishes. The culture medium was refreshed every 2–3 days, and the cells were passaged at 80% confluence by trypsinization. Mycoplasma contamination was assessed using CycleavePCR Mycoplasma Detection Kit (CY232; Takara Bio Inc.). The established cell line was free from mycoplasma. The cells were cultured for more than 9 months (15 passages) and used for the following experiments.

### Cell culture

Normal human dermal fibroblasts (CC-2511; Lonza, Basel, Switzerland) were cultured in DMEM with 10% FBS. Normal human epidermal melanocytes (KM-4109; Kurabo Industries Ltd., Osaka, Japan) were maintained in the DermaLife Ma Comp Kit (LMC-LL0039; Kurabo Industries Ltd.). The acral melanoma cell line SM2-1 (kindly provided by Dr. Hiroshi Murata, Nagano Municipal Hospital, Nagano, Japan) was cultured in RPMI1640 (R8758; Sigma-Aldrich Co.) with 10% FBS. The culture medium was refreshed every 2–3 days, and the cells were passaged at 80% confluence by trypsinization.

### Short tandem repeat (STR) analysis

DNA was extracted from the established cell line (passage 16) and the original tumor lesion using the DNeasy Blood and Tissue Kit (69504; Qiagen, Hilden, Germany), following the manufacturer’s instructions. STR analysis was performed by BEX Co., Ltd. (Tokyo, Japan), using the GenePrint10 System (B934 A; Promega, Madison, WI). The match ratio between the two samples was calculated as follows: (number of coincidental peaks × 2)/total number of peaks in both samples. Samples were considered identical if the match ratio exceeded 0.8 [52].

### Whole-exome sequencing (WES)

WES was conducted to compare the genomic features of the established cell line (passage 16) with those of its original tumor lesion. The WES and subsequent analyses were outsourced to Cell Innovator (Fukuoka, Japan), performed using the Agilent SureSelect v7 system (Agilent Technologies, Santa Clara, CA), as outlined in our previous report [39]. To evaluate DNA mutations, representative cancer-related genes listed in a cancer panel of FoundationOne^®^ CDx (Foundation Medicine Inc., Cambridge, MA) were selected.

### Ploidy analysis

Ploidy of the established cell line was analyzed by flow cytometry. Cells were fixed with 70% ethanol for 30 min on ice and suspended in propidium iodide (2 μg/mL, P3566; Invitrogen) diluted with DPBS. Cells were then analyzed using BD FACSAria Fusion (BD Biosciences, Franklin Lakes, NJ). Normal human epidermal melanocytes were analyzed simultaneously as the control cells of diploid. Results were analyzed with FlowJo software (Tree Star, San Carlos, CA). DNA index (DI) was calculated as the ratio of mean fluorescence intensity of G0/G1 peak in tumor cells and in normal melanocytes. Ploidy was defined based on DI as follows: diploid, DI = 0.95–1.05; hypodiploid, DI < 0.95; hyperdiploid, DI > 1.05–1.92; Tetraploid, DI > 1.92–2.04; hypertetraploid, DI > 2.04; and multiploidy, DI ≥ 2.

### Karyotyping

To investigate the chromosomal abnormalities of the established cell line, karyotyping with a G-band method was performed by Nihon Gene Research Laboratories Inc. (Sendai, Japan). Total of 50 cells were analyzed for its karyotype. Cells at passage 31 were used.

### Cell proliferation curve and cell doubling time

Cell proliferation curve of the cell line was obtained as described in our precious report [39, 40]. The cell doubling time was calculated as follows: ln(2)/[ln(*N*_*t*_/*N*_*0*_)/t], where *N*_*0*_ is the cell number at time 0 and *N*_*t*_ is the cell number at time *t*.

### RNA extraction and reverse-transcription polymerase chain reaction (RT-PCR)

RNA was extracted from the cells using the RNeasy Mini Kit (74104; Qiagen). RT-PCR and following electrophoresis were performed using the PrimeScript RT-PCR Kit (RR014; Takara Bio Inc.) as described in our previous report [39]. The sequences of the primers used are summarized in Supplementary Table S2. β-Actin (*ACTB*) was used as an internal control. RNAs of the acral melanoma cell line SM2-1 and fibroblasts were used as positive and negative controls for melanoma marker expression, respectively. A no-template control (NTC) was also prepared to identify any non-specific amplification.

### Quantitative RT-PCR (qRT-PCR)

qRT-PCR was performed as described in our previous report [39, 40]. *ACTB* was used as an internal control and the relative expression of each gene compared with that of the melanocytes was calculated by the comparative Ct method. The sequences of primers used are summarized in Supplementary Table S2.

### Western blotting

Protein was extracted from the cells and used for western blotting, as detailed in our previous reports [39, 40]. The antibodies used are summarized in Supplementary Table S1. The resulting bands were detected using Super Signal West Pico Chemiluminescent Substrate (34580; Thermo Fisher Scientific, Waltham, MA), and images were captured with the ChemiDoc XRS Plus System (Bio-Rad Laboratories, Hercules, CA). The signals of the bands were quantified using ImageJ software (National Institutes of Health, Bethesda, MD).

### Immunocytochemistry

Cells were seeded into eight-well μ-slides (80826; ibidi GmbH, Gräfelfing, Germany, 10,000 cells/well) and incubated for 2 days at 37 °C in 5% CO_2_. Immunocytochemistry was performed as described in our previous report [39, 40]. The antibodies used are summarized in Supplementary Table S1. Stained cells were covered with mounting medium (H-1200; Vector Laboratories, Burlingame, CA) and observed under an EVOS FL fluorescence microscope (Thermo Fisher Scientific).

### Spheroid formation assay

Cells were seeded into ultra-low-attachment 96-well culture plates (7007; Corning, Corning, NY, 3,000 cells/well) and incubated at 37 °C in a 5% CO_2_ atmosphere. After 72 h of incubation, the formed spheroids were observed under a microscope.

### Invasion assay

The invasiveness of the established cell line was assessed using the Cytoselect 24-well cell invasion assay (CBA-110; Cell Biolabs Inc., San Diego, CA) using a method described in our previous report [39]. The invasive cells on the membrane were stained with a staining solution provided by the kit and examined under a microscope (Nikon Co.).

### Migration assay

Cells were seeded into 96-well IncuCyte Image Lock Plates (4379; Essen Biosciences, Ann Arbor, MI) precoated with collagen type I (Nitta Gelatin Inc., Osaka, Japan) at a cell density of 20,000 cells/well and incubated at 37 °C in a 5% CO_2_ atmosphere for 46 h. Cells were then treated with PBS (vehicle control) or mitomycin C (1.25 μg/mL, MMC, 139–18711; Fujifilm Wako Pure Chemicals) for 2 h and scratched using a wound maker (Essen Biosciences). The images of the scratched cells were captured for 24 h with 2 h interval using the IncuCyte Imaging System (Essen Bioscience). Wound area of each time point relative to that of 0 h was calculated and shown as relative wound area.

### Chemosensitivity and IC_50_

Cells were seeded into 96-well plates (5,000 cells/well) and incubated at 37 °C in a 5% CO_2_ atmosphere for 24 h. The cells were treated with various concentrations of dacarbazine (D3634; Tokyo Chemical Industry Co., Tokyo, Japan), carboplatin (033–25231; Fujifilm Wako Pure Chemicals), and paclitaxel (163–28163; Fujifilm Wako Pure Chemicals), the anticancer drugs used for acral melanoma. Dacarbazine and paclitaxel was dissolved in dimethyl sulfoxide (DMSO, 07–4860-5; Sigma-Aldrich Co.) and carboplatin was dissolved in distilled water. The concentrations of the drugs were determined based on the plasma concentrations of each drug [53]. After 72 h of incubation, viable cells were quantified using CCK-8 solution. The IC_50_ value was calculated using GraphPad Prism 7 software (GraphPad Software, San Diego, CA).

### Statistical analysis

Experiments were conducted multiple times, and the quantitative results are presented as mean ± standard deviation from at least three independent experiments. Statistical analyses were performed using GraphPad Prism 7 software. The significance of differences between two independent groups was assessed using Student’s unpaired two-tailed *t*-test, with *p* < 0.05 considered statistically significant.

## Results

### Clinical features and immunohistochemistry of a patient with NAM

**Fig. 1:**
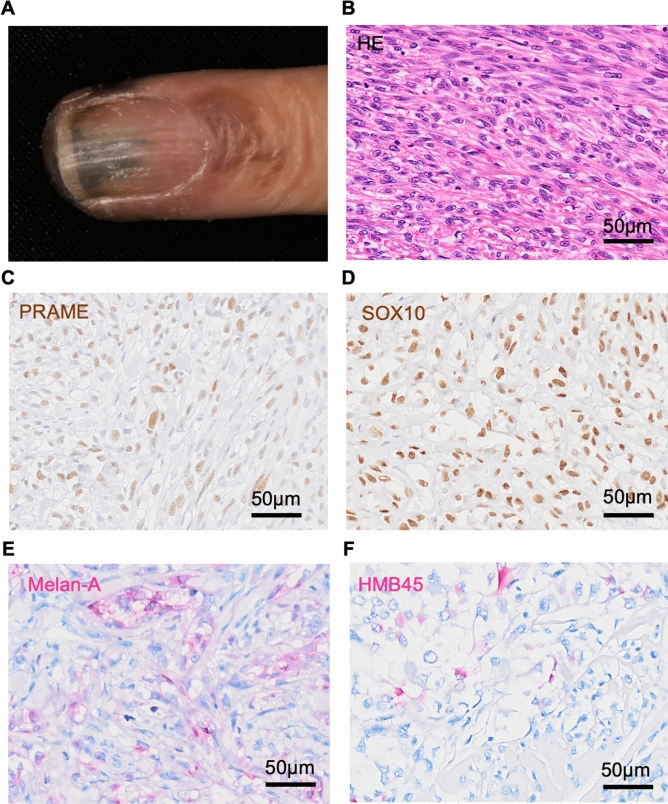
Clinical feature and immunohistochemistry of patient’s melanoma tissue. **A** Macroscopic observation of patient’s melanoma lesion on a nail apparatus. **B** Hematoxylin and eosin staining of patient’s melanoma lesion. **C**–**F** Immunohistochemical images of **C** PRAME, **D** SOX10, **E** HMB45, and **F** Melan-A in the melanoma lesion. Scale bars = 50 μm

The clinical and immunohistochemical features of a melanoma lesion (T4aN1aM0, Stage IIIC) of a 68-year-old Japanese female patient were examined. Clinical findings revealed a subungual black nodule on the left ring finger with a brown patch spreading out from the nail (Fig. 1A). Hematoxylin and eosin staining showed atypical melanocytic cells proliferating in the nail bed epithelium, dermis, and subcutis, focally invading into the bone. Melanin deposition was only noted in tumor cells within or adjacent to the nail bed epithelium. Figure 1B shows a high-power view of tumor cells having no melanin deposition. Expression of several melanoma markers was evaluated by immunohistochemistry. Since the positivity of the markers varies among patients, it is important to combine several markers to accurately characterize the tumor. Here, the expression of PRAME, SOX10, Melan-A, and HMB45 which are routinely used for melanoma diagnosis were evaluated. Immunohistochemically, the tumor cells were diffusely positive for PRAME (Fig. 1C) and SOX10 (Fig. 1D), focally positive for Melan-A (Fig. 1E), and very focally positive for HMB45 (Fig. 1F).

### Characteristics of KS-NailMel-1, a novel cell line derived from a patient’s NAM lesion

**Fig. 2:**
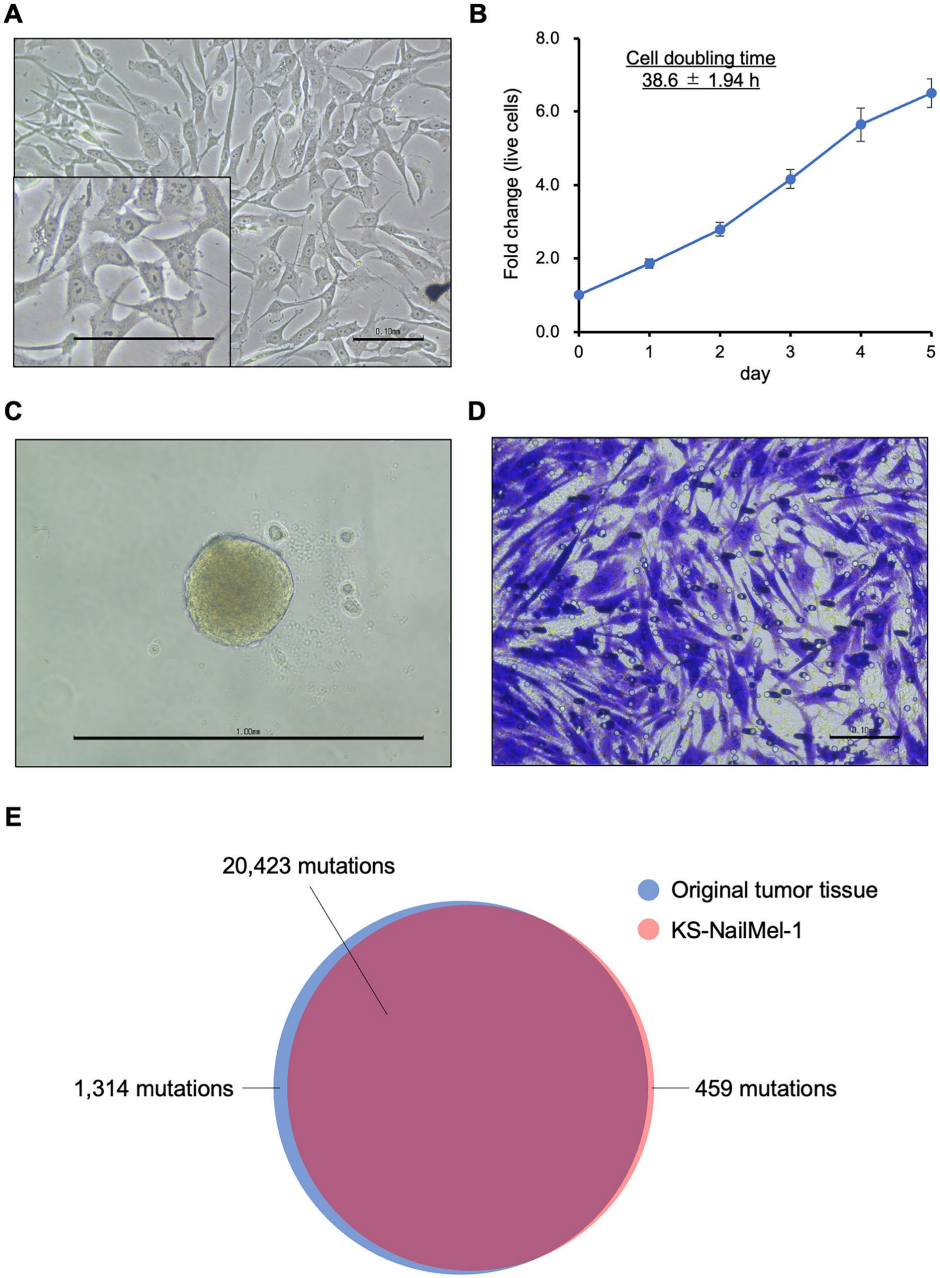
Characteristics of KS-NailMel-1 cells. **A** Morphology of KS-NailMel-1 cells. Scale bars = 100 μm. **B** Cell proliferation curve of the KS-NailMel-1 cells. The cell doubling time was 38.6 ± 1.94 h calculated from three independent experiments. Mean ± standard deviation of fold change of the viable cell number compared with that on day 0 is shown. **C** A representative image of a spheroid formed in an ultra-low-attachment culture plate. Experiments were independently performed three times. Scale bar = 1.0 mm. **D** A representative image of KS-NailMel-1 cells with invasive ability. Cells that passed through the extracellular matrix-coated membrane were stained. Experiments were independently performed three times. Scale bar = 0.1 mm. **E** Whole-exome sequencing comparing mutations in the original tumor lesion and in KS-NailMel-1 cells. There were 20,423 mutations in common between the original tumor lesion and the cell line. Cells at passages 15–20 were used for the experiments in Fig. 2A

A novel cell line, KS-NailMel-1, was established from a NAM lesion of the patient. The cells were stellate or polygonal in shape with round to oval nuclei containing obvious nucleolus (Fig. 2A). The cells grew constantly with a cell doubling time of 38.6 ± 1.94 h (Fig. 2B). Features of the tumor cells such as spheroid-forming ability and invasiveness were also assessed. The KS-NailMel-1 cells formed round and compact spheroids when cultured in ultra-low-attachment culture dishes, indicating they have an ability of anchorage-independent growth (Fig. 2C). Invasion assay revealed that the KS-NailMel-1 cells cultured on the extracellular matrix-coated membrane could pass through it, showing that they possess invasive ability (Fig. 2D). The invasiveness was compared with that of another melanoma cell line SM2-1. A few SM2-1 cells passed through the membranes; however, the number of invaded cells was significantly lesser than that of KS-NailMel-1 cells (Supplementary Fig. S1A, B). Expression of E-cadherin and vimentin, the well-known markers of EMT, was further assessed. Both mRNA and protein expression of E-cadherin (gene symbol; *CDH1*) was significantly lower in KS-NailMel-1 cells compared to that of SM2-1 cells (Supplementary Fig. S1C, D, and S2). On the other hand, *VIM* expression was significantly higher in KS-NailMel-1 cells compared to SM2-1 cells, although the difference at protein level did not reach statistical significance (Supplementary Fig. S1C, D, and S2). Migration assay was also performed and KS-NailMel-1 cells showed significantly slower migration compared with that of SM2-1 cells. MMC was further used to inhibit cell proliferation to avoid it affecting the results. The inhibition of cell proliferation did not change the tendency although the gap between KS-NailMel-1 and SM2-1 cells became narrow (Supplementary Fig. S1E).

### Ploidy and karyotype of KS-NailMel-1

Ploidy assay and karyotyping were further performed to evaluate the DNA contents and abnormality of the chromosome of the KS-NailMel-1 cells. Ploidy assay showed that KS-NailMel-1 cells contain significantly higher amounts of DNA compared to that of normal melanocytes. The DI of KS-NailMel-1 was 1.86 ± 0.307, the value categorized as hyperdiploid (Supplementary Fig. S3A). In agreement with the result of ploidy assay, karyotyping revealed that KS-NailMel-1 cells contain 82–86 chromosomes per a cell (Supplementary Fig. S3B), the number about 1.8-fold higher than that of diploid (46 chromosomes). Additional chromosomes and structural abnormalities were widely observed. In summary, the observed abnormalities are as follows; − X, − X, + add(1)(p11), i(1)(q10), − 2, i(2)(q10), + 3, − 4, − 4, add(5)(p13) × 2, − 6, + 8, add(8)(p11.2), add(8)(p11.2) × 2, add(8)(p11.2) × 2, add(8)(p21), add(9)(p13) × 2, add(10)(q22) × 2, − 11, − 11, add(12)(q24.1) × 2, − 13, − 13, − 15, − 15, add(16)(q22) × 4, − 17, − 18, − 18, add(20)(p13) × 2, − 21, + mar1 × 2, + mar2 × 2.

### KS-NailMel-1 possesses genomic features identical to those of its original tumor lesion

To confirm that the KS-NailMel-1 cells are derived from the original tumor tissue, their genomic features were compared. For this, STR analysis was used as it enables evaluation of the genomic identity between two samples. Allele data are summarized in Table 1. Since the STR profiles of the KS-NailMel-1 cells and its original tumor lesion completely matched (match ratio = 1.0), they were considered to be identical. WES analysis was performed to further compare gene mutations. A total of 20,882 mutations were found in exons of the KS-NailMel-1 cells, 97.8% of which matched with the original lesion (Fig. 2E). Detailed analysis revealed various mutations causing amino acid changes in cancer-related genes such as *EGFR*, *ERBB2*, and *TP53* (Supplementary Table S3). These results suggested that the KS-NailMel-1 cell line has genetic features identical to those of the original tumor.

**Table 1:** STR profiles of KS-NailMel-1 and its original tumor lesion

Locus	KS-NailMel-1		Original tumor lesion	
TH01	9.3		9.3	
D21S11	30	33.2	30	33.2
D5S818	10	12	10	12
D13S317	8	9	8	9
D7S820	8	11	8	11
D16S539	9	10	9	10
CSF1PO	10	12	10	12
AMEL	X		X	
vWA	16	17	16	17
TPOX	11		11	
Match ratio = 1.0				

### Expression of melanoma markers in the KS-NailMel-1 cell line

**Fig. 3:**
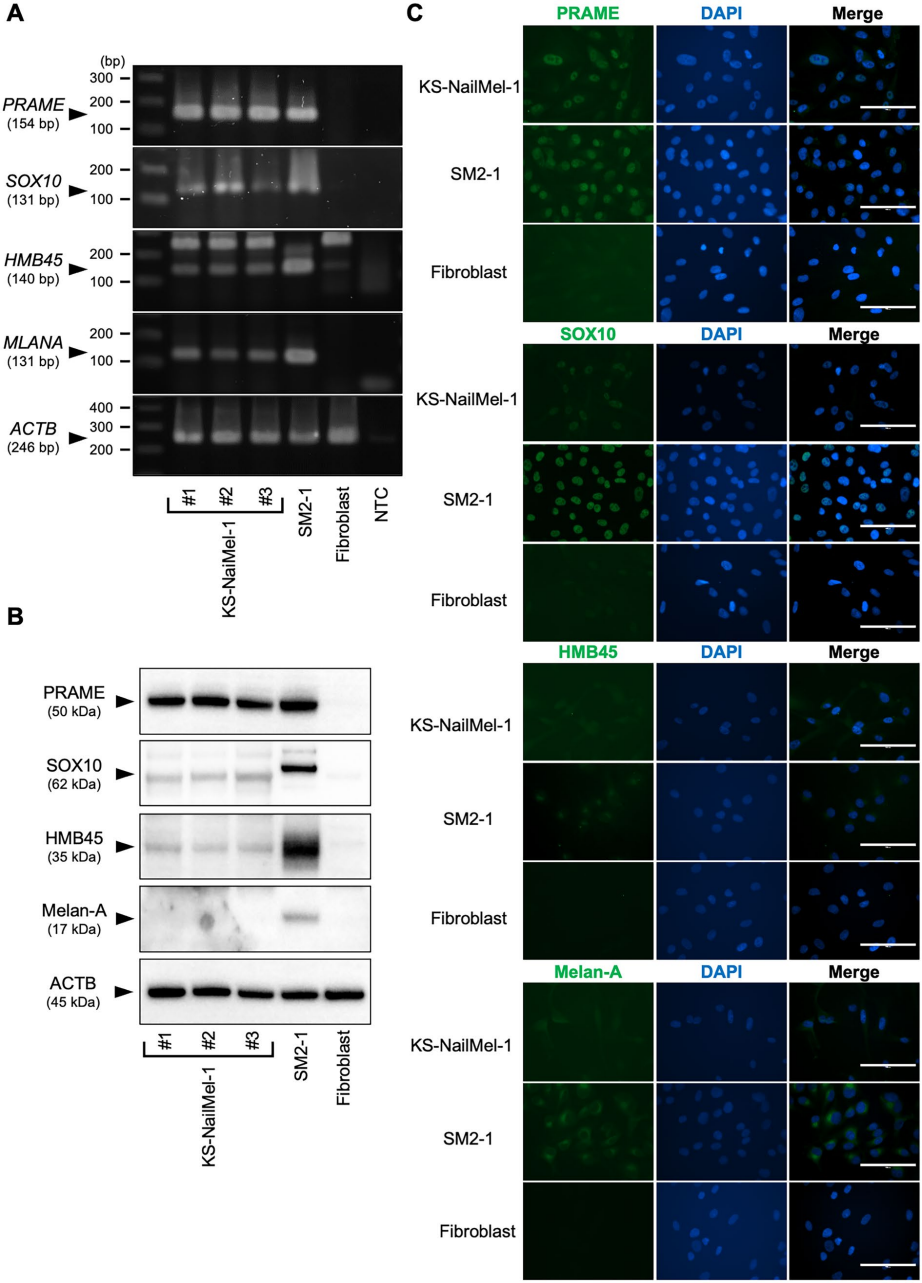
Expression of melanoma markers in KS-NailMel-1 cells. **A** Expression of melanoma marker genes was assessed by RT-PCR. PCR products were run on 2% agarose gels, stained by SYBRGreen I, and visualized using a gel imaging device. #1–3: RNA samples from KS-NailMel-1 cells prepared independently, NTC: no-template control, bp: base pair. Original, full-length images are shown in Supplementary Figure S4. **B** Protein expression of melanoma markers was assessed by western blotting. #1–3: Protein samples from KS-NailMel-1 cells prepared independently. Original, uncropped blot images are shown in Supplementary Figure S5. **C** Representative immunocytochemical images of melanoma markers in KS-NailMel-1, SM2-1, and dermal fibroblasts. Images of isotype control are shown in Supplementary Figure S6. Scale bars = 100 μm. Cells at passages 15–20 were used for the experiments in Fig. 3

To confirm that KS-NailMel-1 cells exhibits feature of melanoma cells, mRNA expression of melanoma markers was evaluated by RT-PCR. An acral melanoma cell line SM2-1 and fibroblasts were used as positive and negative controls. SM2-1 and KS-NailMel-1 cells were positive for *PRAME*, *SOX10*, *HMB45*, and Melan-A (*MLANA*), whereas fibroblasts showed negative (*PRAME*, *SOX10*, and *MLANA*) or faint (*HMB45*) expression (Fig. 3A, Supplementary Fig. S4). Protein expression of these markers was also assessed. KS-NailMel-1 cells were strongly positive for PRAME and weakly positive for HMB45 and SOX10 proteins, but negative for Melan-A (Fig. 3B, Supplementary Fig. S5). Cellular localization of these markers was further evaluated. KS-NailMel-1 and SM2-1 showed nuclear expression of PRAME and SOX10, while fibroblasts were negative for both. Moreover, HMB45 and Melan-A were positive in the cytoplasm of SM2-1, weakly positive in KS-NailMel-1 cells, and negative in fibroblasts (Fig. 3C, Supplementary Fig. S6).

### Gene expression pattern of the KS-NailMel-1 cell line

**Fig. 4:**
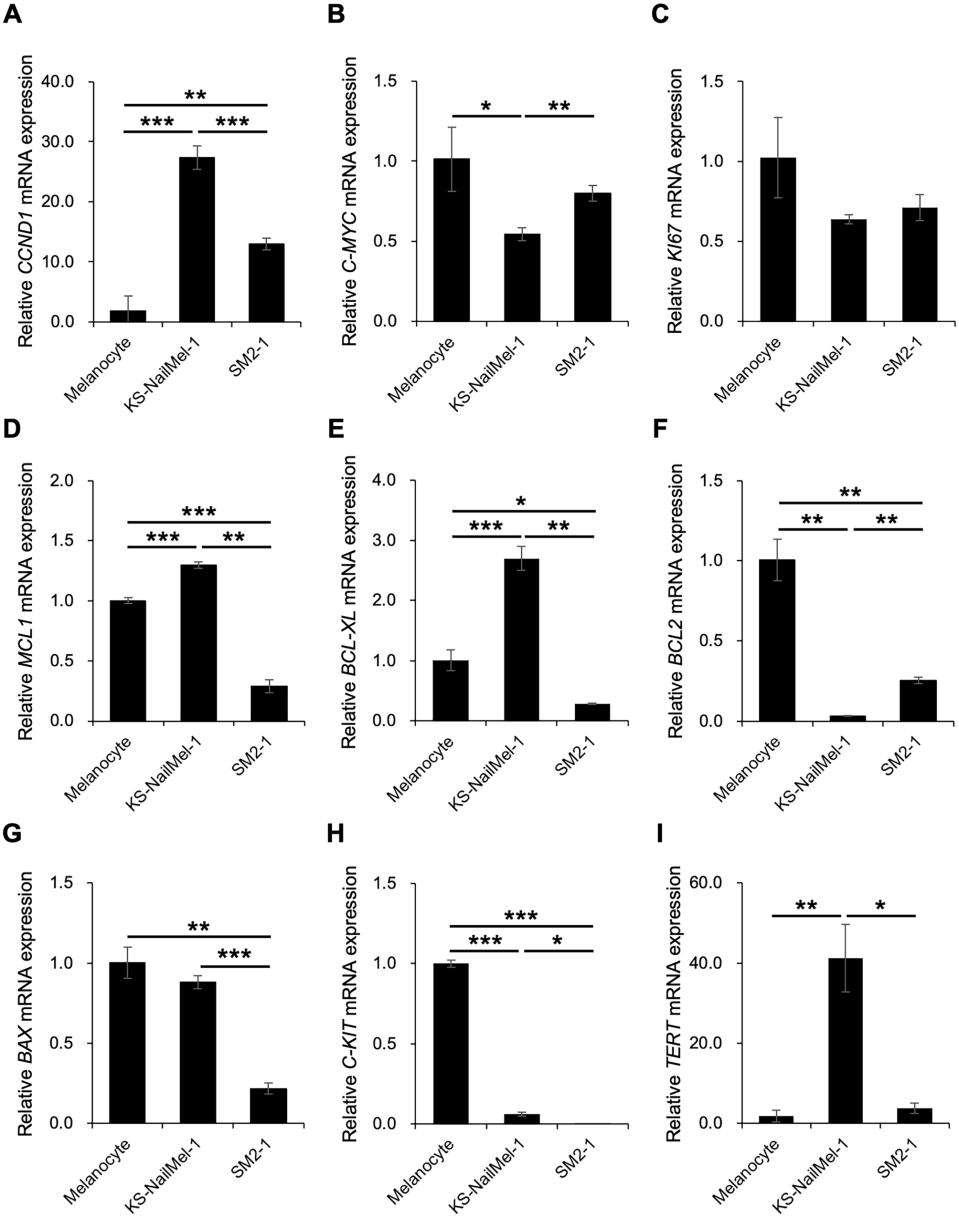
Gene expression patterns of KS-NailMel-1 cells. Gene expression of **A** *CCND1*, **B** *C-MYC*, **C** *KI67*, **D** *MCL1*, **E** *BCL-XL*, **F** *BCL2*, **G** *BAX*, **H** *C-KIT*, and **I** *TERT* in normal melanocytes, KS-NailMel-1, and SM2-1 cells determined by qRT-PCR. Mean ± standard deviation of gene expression relative to that in normal melanocytes obtained from three independent experiments is shown. Cells at passages 15–20 were used. *p < 0.05, **p < 0.01, and ***p < 0.001

To further characterize the KS-NailMel-1 cells, the expression of proliferation-, apoptosis-, and cancer-related genes was assessed (Fig. 4). Melanocytes and SM2-1 cells were assessed simultaneously to compare the expression with that of normal and malignant melanocytes*. CCND1*, a cell cycle regulator, was significantly highly expressed in KS-NailMel-1 and SM2-1 cells compared with that in melanocytes (Fig. 4A). Meanwhile, *C-MYC* and *KI67* showed lower expression in KS-NailMel-1 cells than in melanocytes (Fig. 4B, C). KS-NailMel-1 cells showed significantly higher expression of *MCL1* and *BCL-XL* than did melanocytes and SM2-1 cells (Fig. 4D, E), whereas significantly lower *BCL2* expression than in melanocytes (Fig. 4F). There was no significant difference in expression of the apoptotic gene *BAX* between melanocytes and KS-NailMel-1 cells (Fig. 4G). c-KIT is a receptor of stem cell factor regulating the proliferation and differentiation of melanocytes. *c-KIT* was significantly downregulated in KS-NailMel-1 compared to melanocytes (Fig. 4H). Telomerase reverse transcriptase (*TERT*) is a ribonucleoprotein polymerase maintaining telomere length and abnormal telomerase activation is known to cause immortalization of cancer cells [54]. *TERT* amplification has been observed in acral melanoma at a higher rate than in other types of cutaneous melanoma [55, 56]. In agreement with this, *TERT* was significantly upregulated in the KS-NailMel-1 cells compared with the levels in melanocytes and SM2-1 cells (Fig. 4I). Although *TERT* was highly expressed in KS-NailMel-1 cells, analysis of WES data revealed that there was no mutation identified in *TERT* of KS-NailMel-1 cells.

### Chemosensitivity of the KS-NailMel-1 cell line to anticancer drug

**Fig. 5:**
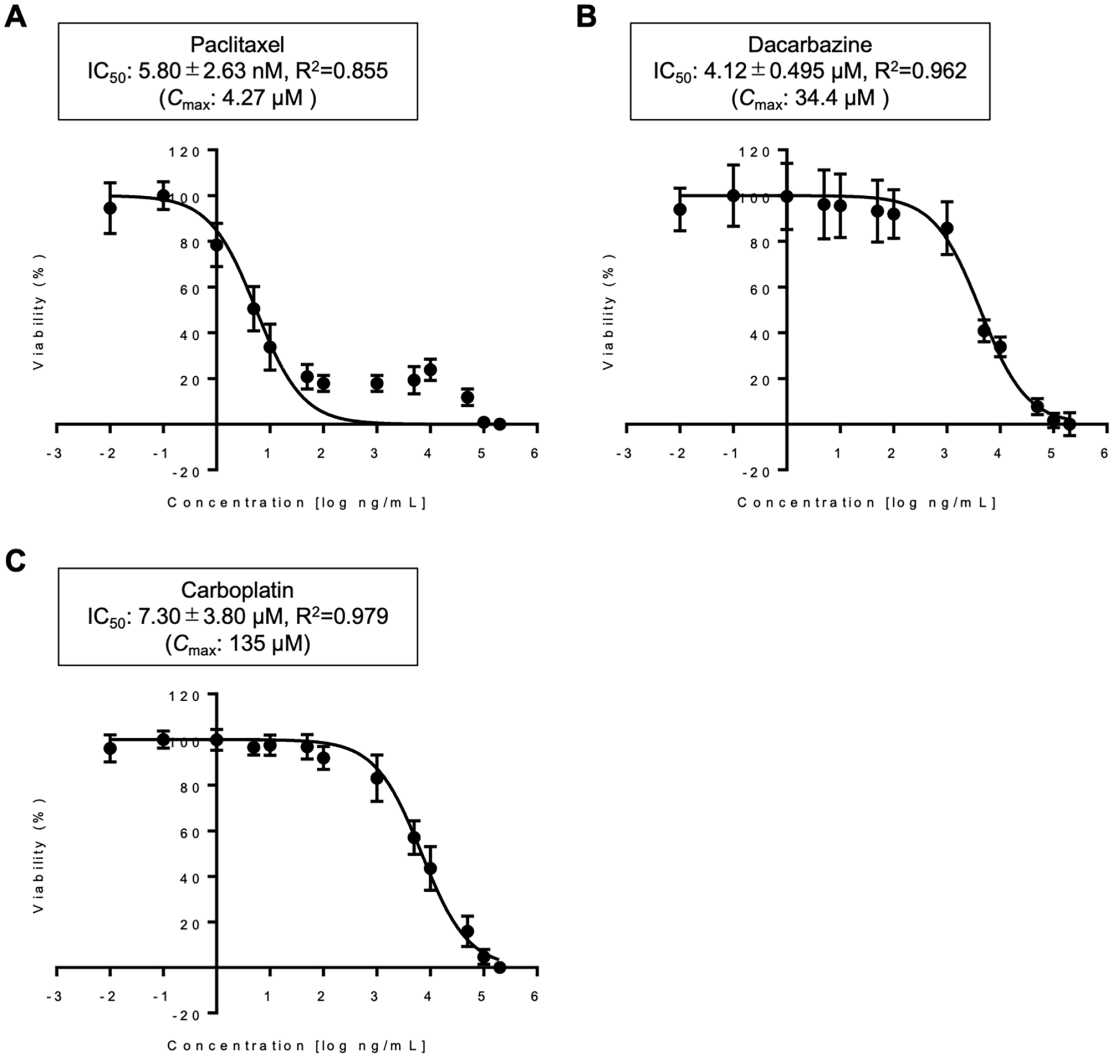
Chemosensitivity of KS-NailMel-1 cells to anticancer drugs. Chemosensitivity of KS-NailMel-1 cells to **A** paclitaxel, **B** dacarbazine, and **C** carboplatin. Experiments were independently repeated three times and mean ± standard deviation of cell viability at 72 h of the treatment is shown. IC_50_ and *C*_max_ of each drug are indicated in the boxes above the graph. Cells at passages 23–25 were used. *p < 0.05, **p < 0.01, and ***p < 0.001

The chemosensitivity of the KS-NailMel-1 cells to anticancer agents used for acral melanoma treatment (i.e., paclitaxel, dacarbazine, and carboplatin) was tested (Fig. 5). Paclitaxel strongly decreased the viability of KS-NailMel-1 cells (Fig. 5A; IC_50_ 5.80 ± 2.63 nM) at a concentration lower than the drug’s maximum concentration in plasma (*C*_max_; 4.27 μM) [53]. Dacarbazine and carboplatin also significantly decreased the cell viability (Fig. 5B and C; IC_50_ 4.12 ± 0.495 μM and 7.30 ± 3.80 μM, respectively) at concentrations lower than their C_max_ (34.4 μM and 135 μM, respectively) [53].

### Expression of NECTIN4 and HER3 in KS-NailMel-1 cell line

**Fig. 6:**
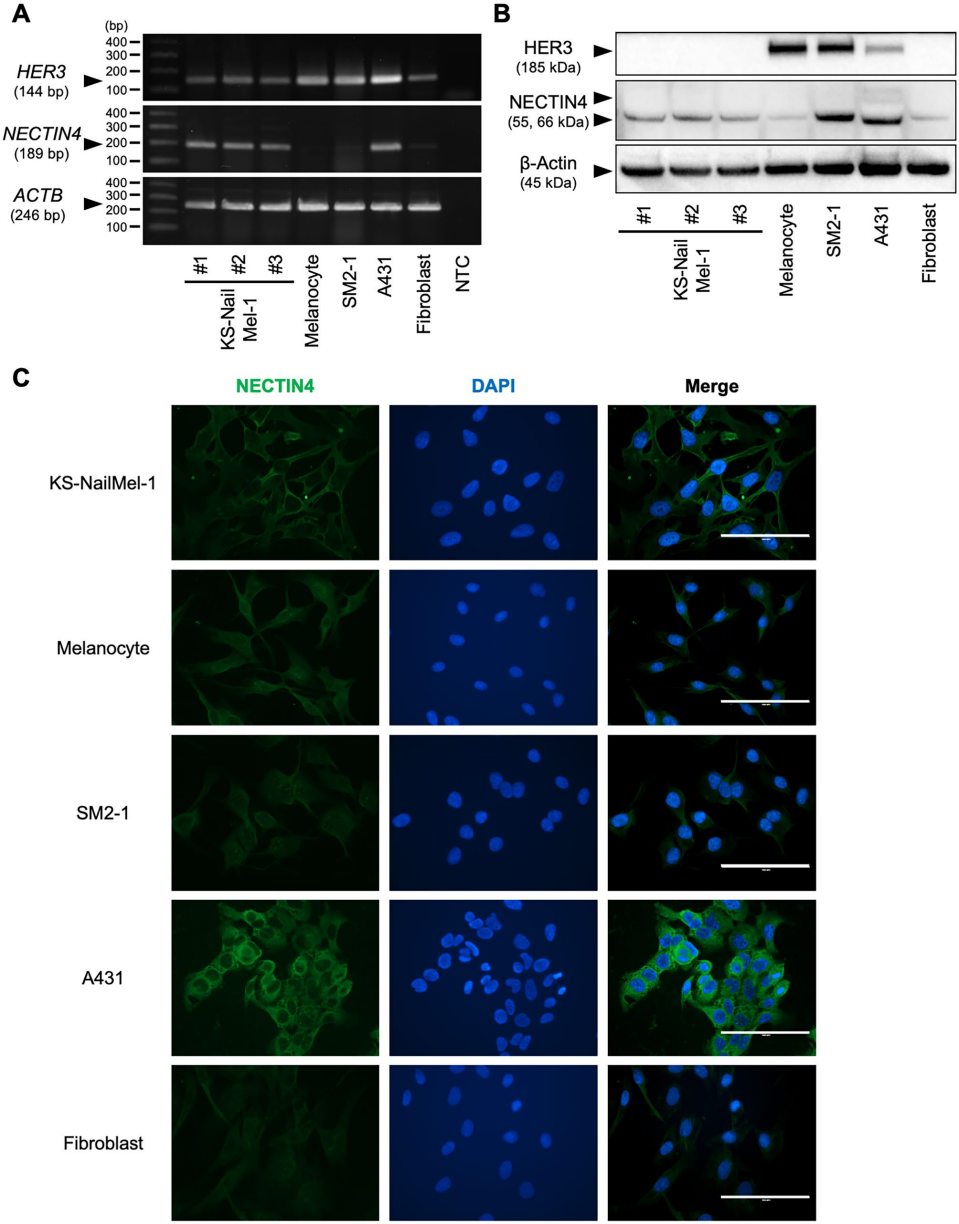
Expression of HER3 and NECTIN4 in KS-NailMel-1 cells. **A** *HER3*, *NECTIN4*, and *ACTB* mRNA expression was assessed by RT-PCR and following electrophoresis. Representative images of electrophoresis are shown. #1−3: RNA samples from KS-NailMel-1 cells prepared independently, NTC: no-template control, bp: base pair. Original, full-length images are shown in Supplementary Figure S7A. **B** HER3, NECTIN4, and β-Actin protein expression was assessed by western blotting. #1−3: protein samples from KS-NailMel-1 cells prepared independently. Original, uncropped blot images are shown in Supplementary Figure S7B. **C** Representative immunocytochemical images of NECTIN4 in KS-NailMel-1, melanocytes, SM2-1, A431, and fibroblasts. Images of isotype control are shown in Supplementary Figure S8. Experiments were independently performed three times. Scale bars = 100 μm. KS-NailMel-1 cells at passage 15−20 were used for the experiments in Fig. 6

HER3 and NECTIN4 are regarded as promising therapeutic targets for various cancers including melanoma [45, 48]. Then, their expression was assessed in KS-NailMel-1 cells. KS-NailMel-1 cells weakly expressed *HER3* mRNA but its protein was undetectable. NECTIN4 was expressed in KS-NailMel-1 cells at both mRNA and protein levels (Fig. 6A and B, Supplementary Fig. S7A, B). Since NECTIN4 protein was detected, its cellular localization was further evaluated. NECTIN4 was observed on membranes and cytoplasm of KS-NailMel-1 cells (Fig. 6C, Supplementary Fig. S8).

## Discussion

NAM is a rare form of melanoma that occurs in the nail apparatus. It is still challenging to effectively treat this disease due to its distinct features and also the limited access to its cancer cell line available for analyses. Here we successfully established a novel NAM cell line from a NAM lesion of a patient and characterized its features. The established cell line exhibited the characteristics of melanoma and showed a unique gene expression pattern compared with melanocyte and other melanoma cells. The established cell line also enabled to assess the toxicity of anticancer drugs. Our results suggest that the newly established NAM cell line represents a promising resource for various researches on NAM.

Interest in acral melanoma and NAM is growing, but there are still a limited number of acral melanoma cell lines available for research. To date, only one other NAM cell line has been established from a primary NAM lesion, making the KS-NailMel-1 cell line reported here the second of its kind [41]. The XYAM-2 cell line was derived from a primary NAM lesion on the right thumb. Exome sequencing revealed that the XYAM-2 cell line was negative for mutations in major melanoma driver genes, including *BRAF*, *NRAS*, and *NF1*. Similarly, our WES analysis revealed that the KS-NailMel-1 cells are negative for mutations in these genes (Supplementary Table S3). Of note, XYAM-2 cells and KS-NailMel-1 cell lines both have mutation in the exon of a tumor suppressor gene *APC*. However, other detailed features and characteristics of XYAM-2 cells are not explained well in the report [41], thus our newly established NAM cell line and investigation on them would give useful information for the future analyses on NAM. A recent study found that triple-wild-type melanomas, which lack mutations in the three major melanoma-related genes, were associated with significantly shorter survival when treated with ICIs [57]. This aligns with the challenges faced in immunotherapy for NAM, in which mutations in the three major genes are uncommon.

We further compared expression of various cancer-related genes in normal melanocytes and a well-known acral melanoma cell line SM2-1 (Fig. 4). Higher expression of *CCND1*, *MCL1*, *BCL-XL*, and *TERT* in KS-NailMel-1 cells implied the enhanced growth and/or survival of the cells. Analysis of WES data revealed that there was no mutation identified in *TERT* of KS-NailMel-1 cells, implying the post-transcriptional regulation may cause the upregulation of *TERT*. Looking at the invasiveness of the cells, KS-NailMel-1 cells seem to show increased invasiveness than that of SM2-1 cells accompanied with decreased E-cadherin and increased vimentin expression (Supplementary Fig. S1, 2). SM2-1 cells were derived from acral lentiginous melanoma with in-transit metastasis in hypodermis, whereas our patients’ NAM lesions invaded the bone. It can be estimated that the invasive/metastatic status of the original tumor lesion may affect the invasive nature of the cell lines, however it has not been clearly explained yet. Thus, in vivo metastasis assay would be performed in the future study to more accurately explain the invasive nature of the KS-NailMel-1 cells. As explained above, KS-NailMel-1 and SM2-1 cells showed quite different gene expression profiles and invasiveness, although both cell lines belong to acral melanoma. As acral melanoma cell lines show heterogeneous features [41], using multiple cell lines with various origins is ideal. However, the number of the acral melanoma cell lines, especially NAM-derived, is limited and the cell lines are not widely available at present. Thus, the newly established KS-NailMel-1 cells will contribute to expanding the variety of analyses on acral melanoma.

The KS-NailMel-1 cells were confirmed to be identical to its original tumor through immunostaining, STR analysis, and WES. The original tumor cells exhibited diffuse positivity for PRAME and SOX10, while they were focally positive for Melan-A and very focally positive for HMB45, confirming the diagnosis of melanoma [58–60]. The KS-NailMel-1 cells also demonstrated expression of these markers on immunocytochemistry; however, the expression of SOX10, HMB45, and Melan-A proteins was weak or negative when analyzed by western blotting. The STR profiles of the KS-NailMel-1 cells and its original lesion were completely identical. Additionally, both the cell line and the tumor share over 92% of genetic mutations, which further confirms their identity.

We previously found that target antigens of various antibody–drug conjugates (ADCs) were expressed in various skin tumors including melanoma [42–49, 61, 62]. ADCs are emerging therapeutics consisting of a monoclonal antibody linked to a cytotoxic agent via a linker, allowing specific delivery of the cytotoxic agent to target cells. In the current study, we examined the expression of HER3 and NECTIN4, which we previously assessed in melanoma. We found that HER3 was negative, while NECTIN4 was positive. We first assumed that HER3 might be strongly expressed in the KS-NailMel-1 cell line, but not. Considering that HER3 expression level varies among acral melanoma patients [48] and some patients were negative for HER3, it seemed that our newly established cell line originated from a HER3-negative fraction of the tumor cells. ADCs targeting these proteins are already used clinically for some cancers [63] and it could represent a novel treatment option for NAM. Although it is ideal to perform further validation experiments using in vivo tumorigenic model, the in vivo xenograft model of NAM has not yet been established well. Besides, we have limited equipment and resources to establish the model at present. Thus, in vivo assays will be focused in future studies. Our newly established KS-NailMel-1 cell line will serve as a useful tool for the assessment of those therapeutic agents.

In conclusion, we successfully established a NAM cell line, KS-NailMel-1, from a patient’s primary tumor. This cell line demonstrated consistent growth, the ability to form spheroids, and invasiveness in vitro. Further ex vivo analyses are needed to better characterize the tumor and develop effective treatment strategies for this rare cancer. 

## Supplementary Information

Below is the link to the electronic supplementary material.

Supplementary file1 (DOCX 33 KB)

Supplementary file2 (TIFF 10263 KB)

Supplementary file3 (TIFF 10263 KB)

Supplementary file4 (TIFF 10263 KB)

Supplementary file5 (TIFF 10263 KB)

Supplementary file6 (TIFF 10263 KB)

Supplementary file7 (TIFF 10263 KB)

Supplementary file8 (TIFF 10263 KB)

Supplementary file9 (TIFF 10263 KB)

## Data Availability

The data related to this study are included in the article and supplementary materials. Further inquiries can be directed to the corresponding author.
