# Supplementary material for: KS-NailMel-1: a novel cell line of nail apparatus melanoma
Source: Hum Cell. 2025 May 28;38(4):112. doi: 10.1007/s13577-025-01242-7 (PMC12119781; doi:10.1007/s13577-025-01242-7)
Supplement: Supplementary file 1 — Supplementary file1 (DOCX 33 KB) [file 13577_2025_1242_MOESM1_ESM.docx]

**Supplementary Table S1.** Specification of antibodies used for immunohistochemistry, western blotting, and immunocytochemistry.

| Antigen | Host | Manufacturer | Catalog no. | Application (dilution) |
| --- | --- | --- | --- | --- |
| β-Actin | Rabbit | Cell Signaling Technology | 4970 | WB (1:2,000) |
| E-cadherin | Mouse | BD Biosciences | 610181 | WB (1:5,000) |
| HER3 | Rabbit | Santa Cruz Biotechnology | 12808 | WB (1:1,000) |
| HMB45 | Mouse  Mouse | Enzo Life Sciences  Abcam | ENZ-30930  ab787 | IHC (ready-to-use)  WB (1:1,000)  ICC (1:100) |
| Melan-A | Mouse  Mouse | Leica Biosystems  Santa Cruz Biotechnology | NCL-L-MelanA  SC-20032 | IHC (1:25)  WB (1:1,000)  ICC (1:100) |
| NECTIN4 | Rabbit | Abcam | ab235897 | WB (1:1,000)  ICC (1:200) |
| PRAME | Rabbit  Rabbit | Abcam  Cell Signaling Technology | ab219650  56426 | IHC (1:200)  WB (1:1,000)  ICC (1:100) |
| SOX10 | Mouse  Rabbit | Nichirei Biosciences  Cell Signaling Technology | 718241  89356 | IHC (ready-to-use)  WB (1:500)  ICC (1:200) |
| Vimentin | Rabbit | Cell Signaling Technology | 5741 | WB (1:1,000) |
| Mouse/  Rabbit IgG | Goat | Nichirei Biosciences | 724152 | IHC (ready-to-use)  *AP-labeled |
| Mouse/  Rabbit IgG | Goat | Nichirei Biosciences | 725191 | IHC (ready-to-use)  *Peroxidase-labeled amino acid polymer-conjugated |
| Mouse IgG | Goat | Cell Signaling Technology | 7076 | WB (1:10,000)  *HRP-conjugated |
| Rabbit IgG | Goat | Cell Signaling Technology | 7074 | WB (1:10,000)  *HRP-conjugated |
| Mouse IgG | Goat | Thermo Fisher Scientific | A11001 | ICC (1:400)  *AlexaFluor^488^-conjugated |
| Rabbit IgG | Goat | Thermo Fisher Scientific | A11008 | ICC (1:400)  *AlexaFluor^488^-conjugated |

IHC, immunohistochemistry; WB, western blotting; ICC, immunocytochemistry; AP, alkaline-phosphatase; HRP, horseradish peroxidase.

## Supplementary Table S2. Sequences of primers used for RT-PCR and qRT-PCR.

| Gene symbol | Primer sequence |
| --- | --- |
| *ACTB*  (for qPCR) | Sense: 5′-ATTGCCGACAGGATGCAGA-3′ |
|  | Antisense: 5′-GAGTACTTGCGCTCAGGAGGA-3′ |
| *ACTB*  (for RT-PCR) | Sense: 5′-AAGAGCTACGAGCTGCCTGA-3′ |
|  | Antisense: 5′-GGCAGTGATCTCCTTCTGCA-3′ |
| *BAX* | Sense: 5′-GGACGAACTGGACAGTAACATGG-3′ |
|  | Antisense: 5′-GCAAAGTAGAAAAGGGCGACAAC-3′ |
| *BCL2* | Sense: 5′-ATCGCCCTGTGGATGACTGAG-3′ |
|  | Antisense: 5′-CAGCCAGGAGAAATCAAACAGAGG-3′ |
| *BCL-XL* | Sense: 5′-TTACCTGAATGACCACCTA-3′ |
|  | Antisense: 5′-ATTTCCGACTGAAGAGTGA-3′ |
| *CCND1* | Sense: 5′-GCTGCGAAGTGGAAACCATC-3′ |
|  | Antisense: 5′-CCTCCTTCTGCACACATTTGAA-3′ |
| *CDH1* | Sense: 5′-TGCCCAGAAAATGAAAAAGG-3′ |
|  | Antisense: 5′-GTGTATGTGGCAATGCGTTC-3′ |
| *C-KIT* | Sense: 5′-CACCGAAGGAGGCACTTACACA-3′ |
|  | Antisense: 5′-TGCCATTCACGAGCCTGTCGTA-3′ |
| *C-MYC* | Sense: 5′-GAGGCGAACACACAACGTCTT-3′ |
|  | Antisense: 5′-CGCAACAAGTCCTCTTCAGAAA-3′ |
| *HER3* | Sense: 5′-CTATGAGGCGATACTTGGAACGG-3′ |
|  | Antisense: 5′-GCACAGTTCCAAAGACACCCGA-3′ |
| *HMB45* | Sense: 5′-CTGCCTCAATGTGTCTCTGGCT-3′ |
|  | Antisense: 5′-CAAGGACCACAGCCATCAACAC-3′ |
| *KI67* | Sense: 5′-TTGGAGAATGACTCGTGAGC-3′ |
|  | Antisense: 5′-CGAAGCTTTCAATGACAGGA-3′ |
| *MCL1* | Sense: 5′-AACAAAGAGGCTGGGATG-3′ |
|  | Antisense: 5′-ATTGCACTTACAGTAAGGCTATC-3′ |
| *MLANA* | Sense: 5′-GGACAGCAAAGTGTCTCTTCAAG-3′ |
|  | Antisense: 5′-TCAGGTGTCTCGCTGGCTCTTA-3′ |
| *NECTIN4* | Sense: 5′-CAAAATCTGTGGCACATTGG-3′ |
|  | Antisense: 5′-GCTGACATGGCAGACGTAGA-3′ |
| *PRAME* | Sense: 5′-GGAGTGCTGATGAAGGGACAAC-3′ |
|  | Antisense: 5′-CAGTCCAGAAGTCCTGATGAGAG-3′ |
| *SOX10* | Sense: 5′-ATGAACGCCTTCATGGTGTGGG-3′ |
|  | Antisense: 5′-CGCTTGTCACTTTCGTTCAGCAG-3′ |
| *TERT* | Sense: 5′-GCCGATTGTGAACATGGACTACG-3′ |
|  | Antisense: 5′-GCTCGTAGTTGAGCACGCTGAA-3′ |
| *VIM* | Sense: 5′-GAGAACTTTGCCGTTGAAGC-3′ |
|  | Antisense: 5′-GCTTCCTGTAGGTGGCAATC-3′ |

**Supplementary Table S3.** List of mutations found in representative cancer-related genes in original tumor lesion and KS-Mel nail-1 cell line.

| Symbol | Chr | Ref | Alt | Consequence | Amino acid |
| --- | --- | --- | --- | --- | --- |
| ALK | 2 | G  T | C  C | missense_variant  missense_variant | D/E  K/R, I/V |
| APC | 5 | T | A | missense_variant | V/D |
| AR | X | TGGC | T | inframe_deletion | G/− |
| ASXL1 | 20 | G | A | missense_variant | G/S |
| ATR | 3 | A | G | missense_variant | M/T |
| ATRX | X | C | G | missense_variant | E/Q |
| AURKA | 20 | T  A | C  T | missense_variant  missense_variant | I/V  F/I |
| AURKB | 17 | A | G | missense_variant | M/T |
| BARD1 | 2 | C  C  G  G | T  G  C  A | missense_variant  missense_variant  missense_variant  missense_variant | V/M  R/S  S/C  P/S |
| BCL2L2 | 14 | A | G | missense_variant | Q/R |
| BRCA1 | 17 | T  G | C  A | missense_variant  missense_variant | S/G, K/R, E/G  P/L |
| BRCA2 | 13 | A  T | C  C | missense_variant  missense_variant | N/H  V/A |
| BRIP1 | 17 | A | G | missense_variant | S/P |
| BTG2 | 1 | A | T | missense_variant | S/C |
| CDH1 | 16 | C | G | missense_variant | L/V |
| CHEK1 | 11 | A | G | missense_variant | I/V |
| CSF1R | 5 | T | C | missense_& splice_region_variant | H/R |
| CUL4A | 13 | A | G | missense_variant | K/R |
| DIS3 | 13 | G | C | missense_variant | T/R |
| EGFR | 7 | G | A | missense_variant | R/K |
| ERBB2 | 17 | C | G | missense_variant | P/A |
| FANCA | 16 | C  T | T  C | missense_variant  missense_variant | G/D, G/S  T/A |
| FGFR4 | 5 | G  C  A | A  T  G | missense_variant  missense_variant  missense_variant | V/I, G/R  P/L  T/A |
| FLT3 | 13 | G  T | A  C | missense_variant  missense_variant | T/M  D/G |
| GRM3 | 7 | G | T | missense_variant | E/D |
| HNF1A | 12 | A  G  A | C  A  G | missense_variant  missense_variant  missense_variant | I/L  S/N  S/G |
| HSD3B1 | 1 | C | A | missense_variant | T/N |
| ID3 | 1 | T | C | missense_variant | T/A |
| INPP4B | 4 | C | T | missense_variant | G/S |
| IRS2 | 13 | C | T | missense_variant | G/D |
| KDM5A | 12 | A | G | missense_variant | M/T |
| KMT2D | 2 | T  A | C  T | missense_variant  missense_variant | M/V  S/T |
| MAP2K4 | 17 | C | T | missense_variant | A/V |
| MAP3K1 | 5 | TCAA  G | T  A | inframe_deletion  missense_variant | ST/S  V/I |
| MSH2 | 2 | C | T | missense_variant | L/F |
| MSH3 | 5 | A  G | G  A | missense_& splice_region_variant  missense_& splice_region_variant | I/V, Q/R  A/T |
| MST1R | 3 | T | C | missense_variant | N/S |
| MYCL | 1 | G | C | missense_variant | T/S |
| NBN | 8 | C | G | missense_variant | E/Q |
| NOTCH2 | 1 | A | T | missense_variant | I/N |
| NOTCH3 | 19 | G | A | missense_variant | A/V |
| PARP1 | 1 | A | G | missense_variant | V/A |
| PDCD1 | 2 | G | A | missense_variant | A/V |
| PDCD1LG2 | 9 | T | C | missense_variant | F/S |
| PMS2 | 7 | T  G | C  T | missense_variant  missense_variant | K/E  T/K |
| PRDM1 | 6 | C | G | missense_variant | D/E |
| PTCH1 | 9 | G | A | missense_variant | P/L |
| RAD51C | 17 | G | A | missense_variant | R/H |
| RET | 10 | G | A | missense_variant | D/N |
| RICTOR | 5 | G | A | missense_variant | S/F |
| RNF43 | 17 | G | T | missense_variant | L/M |
| SETD2 | 3 | G | A | missense_variant | P/L |
| TEK | 9 | A | C | missense_variant | Q/P |
| TNFRSF14 | 1 | A | G | missense_variant | K/R |
| TP53 | 17 | G | C | missense_variant | P/R |
