# Supplementary figures and images for: KS-NailMel-1: a novel cell line of nail apparatus melanoma
Source: Hum Cell. 2025 May 28;38(4):112. doi: 10.1007/s13577-025-01242-7 (PMC12119781; doi:10.1007/s13577-025-01242-7)

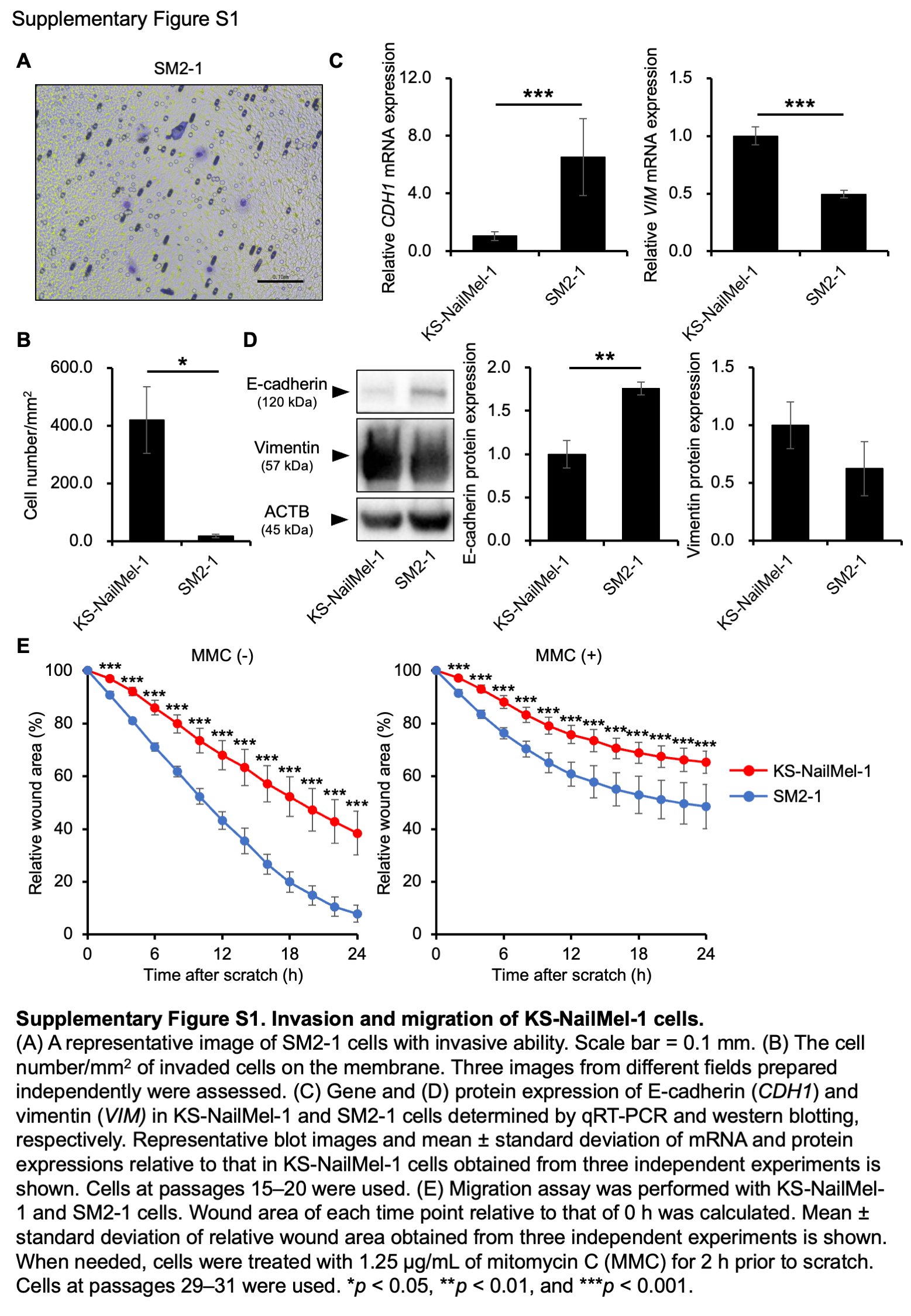

Supplement: Supplementary file 2 — Supplementary file2 (TIFF 10263 KB) [file 13577_2025_1242_MOESM2_ESM.tiff]

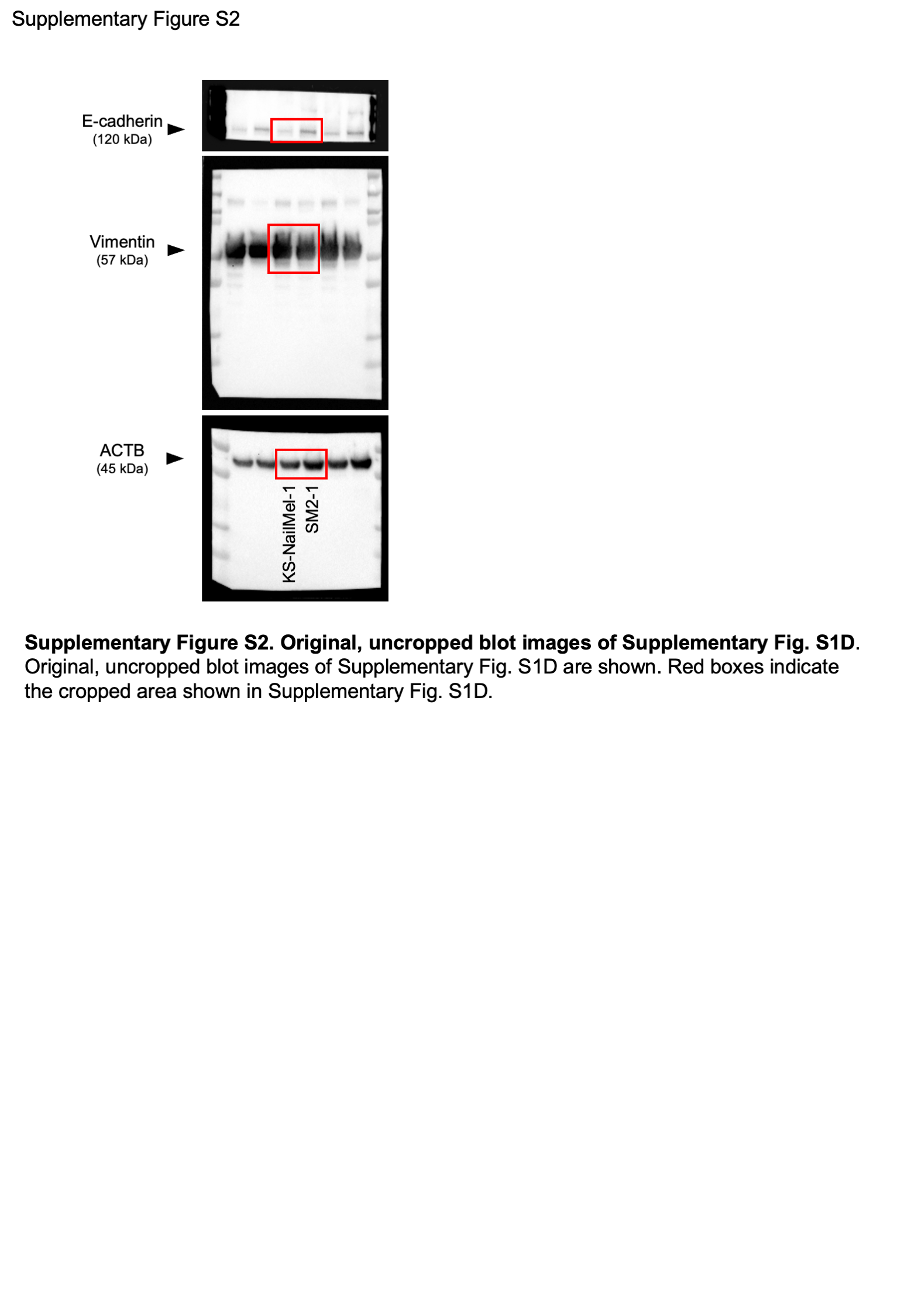

Supplement: Supplementary file 3 — Supplementary file3 (TIFF 10263 KB) [file 13577_2025_1242_MOESM3_ESM.tiff]

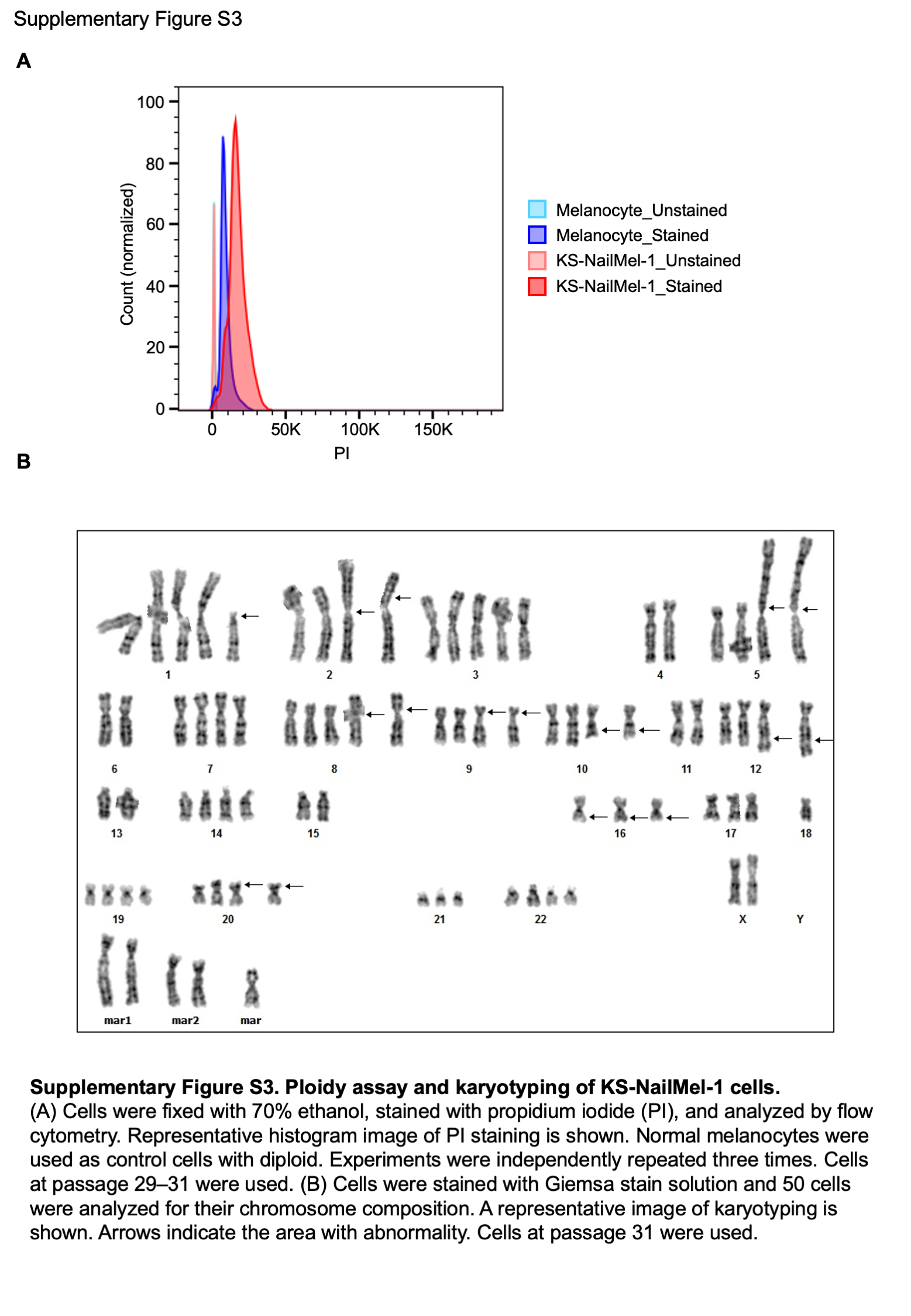

Supplement: Supplementary file 4 — Supplementary file4 (TIFF 10263 KB) [file 13577_2025_1242_MOESM4_ESM.tiff]

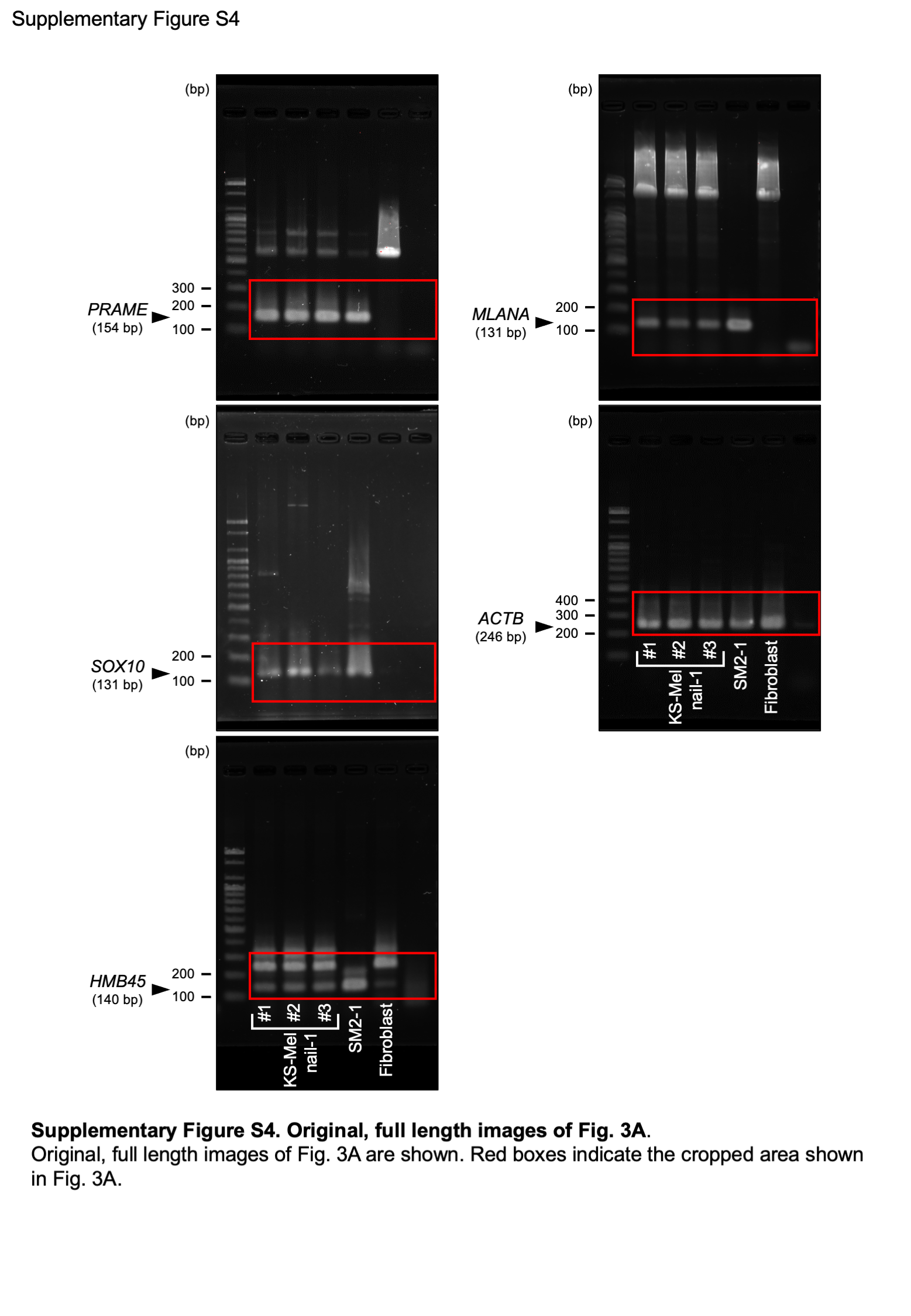

Supplement: Supplementary file 5 — Supplementary file5 (TIFF 10263 KB) [file 13577_2025_1242_MOESM5_ESM.tiff]

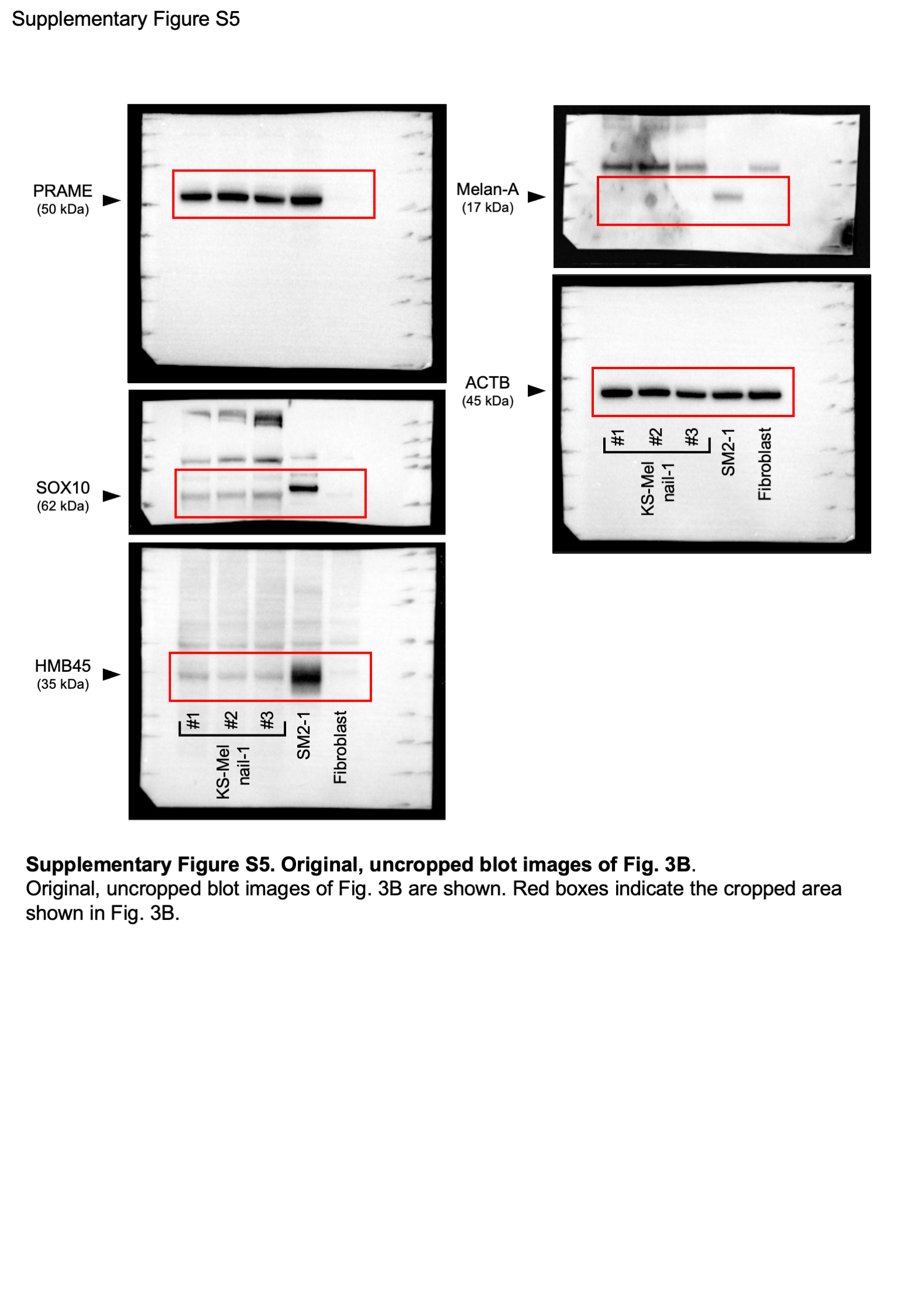

Supplement: Supplementary file 6 — Supplementary file6 (TIFF 10263 KB) [file 13577_2025_1242_MOESM6_ESM.tiff]

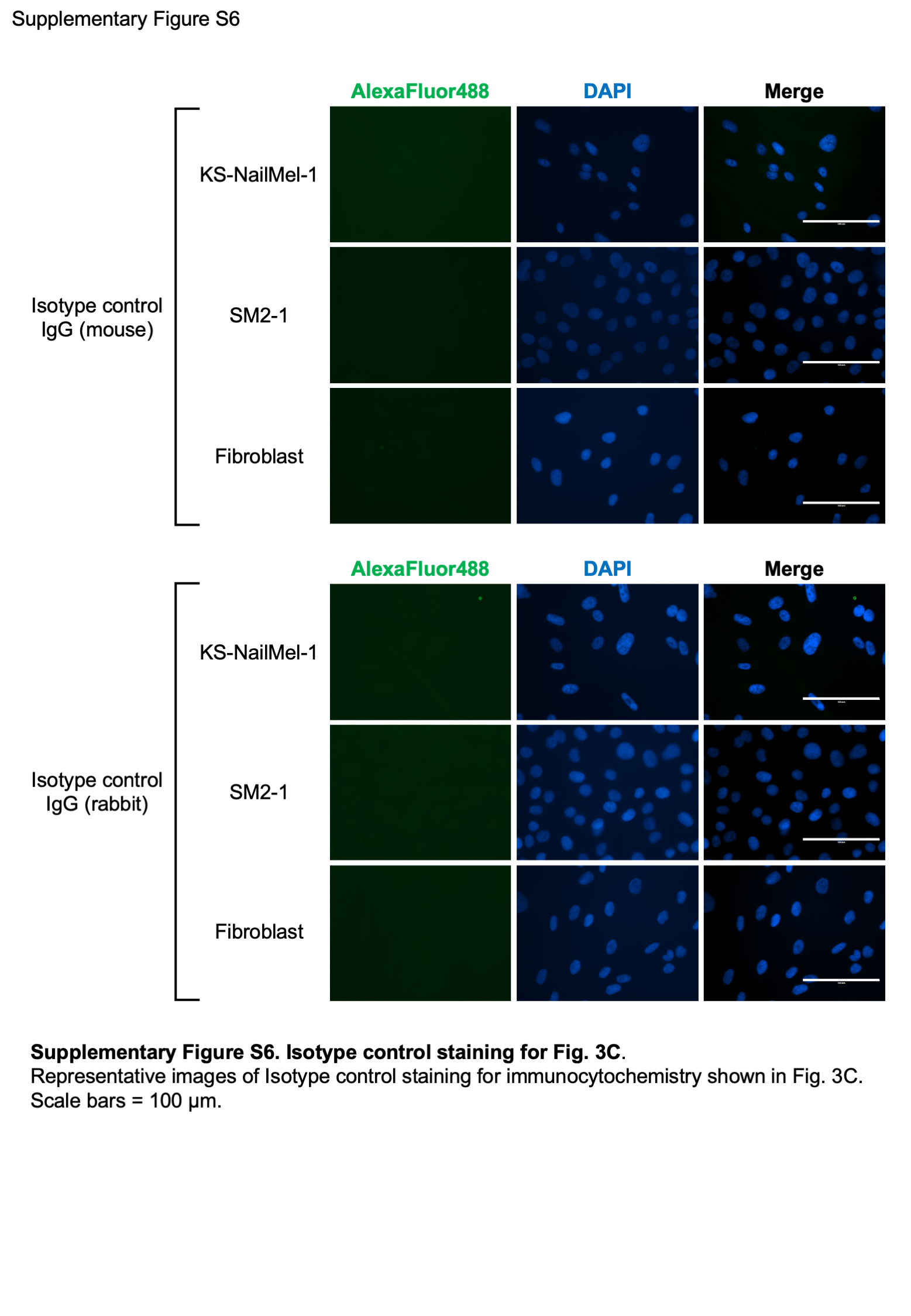

Supplement: Supplementary file 7 — Supplementary file7 (TIFF 10263 KB) [file 13577_2025_1242_MOESM7_ESM.tiff]

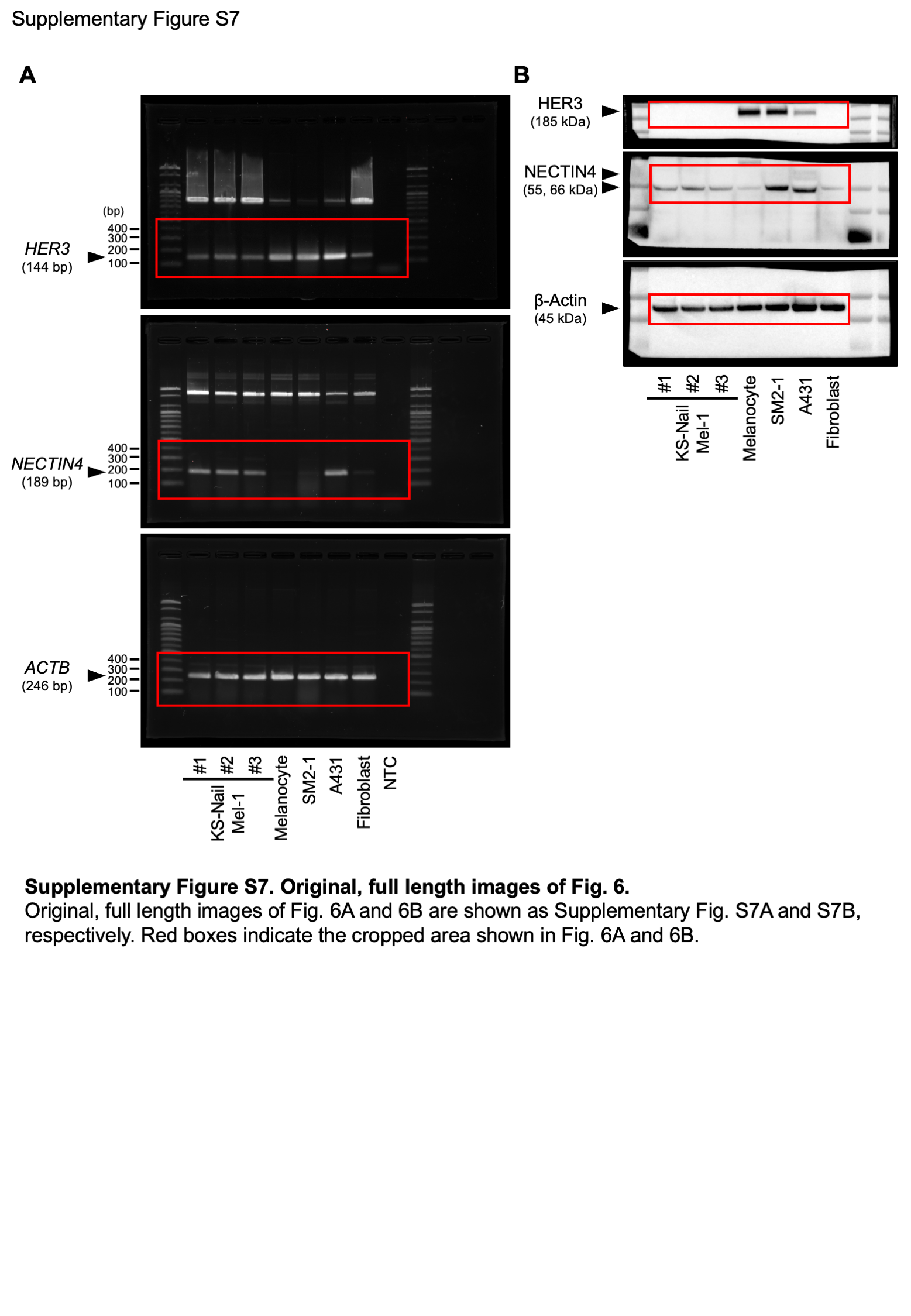

Supplement: Supplementary file 8 — Supplementary file8 (TIFF 10263 KB) [file 13577_2025_1242_MOESM8_ESM.tiff]

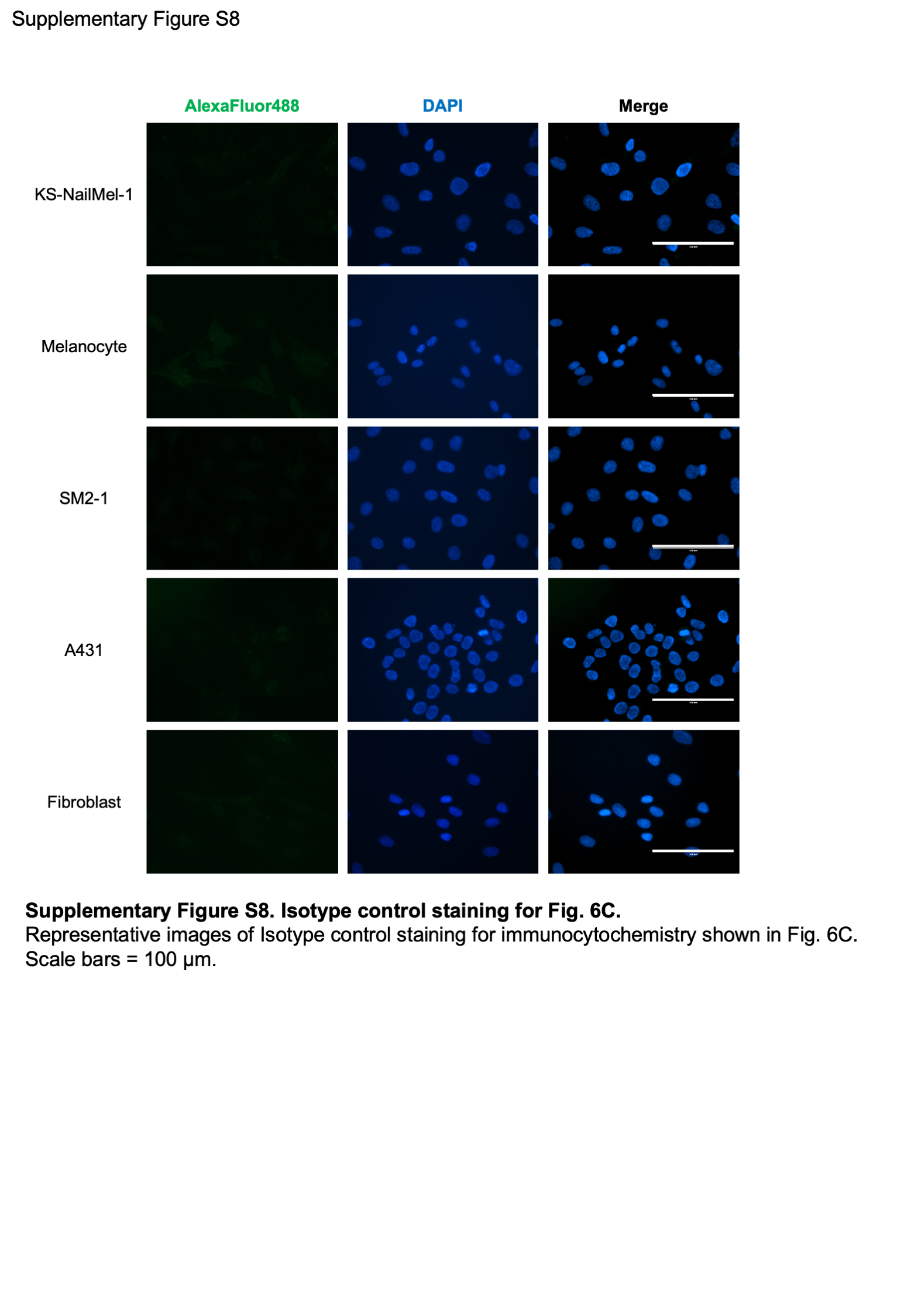

Supplement: Supplementary file 9 — Supplementary file9 (TIFF 10263 KB) [file 13577_2025_1242_MOESM9_ESM.tiff]
